# Supplementary material for: A fully joint Bayesian quantitative trait locus mapping of human protein abundance in plasma
Source: PLoS Comput Biol. 2020 Jun 3;16(6):e1007882. doi: 10.1371/journal.pcbi.1007882 (PMC7295243; doi:10.1371/journal.pcbi.1007882)
Supplement: S1 Appendix — Scatterplots for the 72 protein levels with dual-technology quantification for both the Ottawa and the DiOGenes cohorts. (PDF) [file pcbi.1007882.s001.pdf]

Proteins measured by the MS and SomaLogic technologies

Scatterplots for the 72 proteins having both MS and SomaLogic measurements, with loess fit. Blue plot titles indicate the proteins involved in validated pQTL hits.

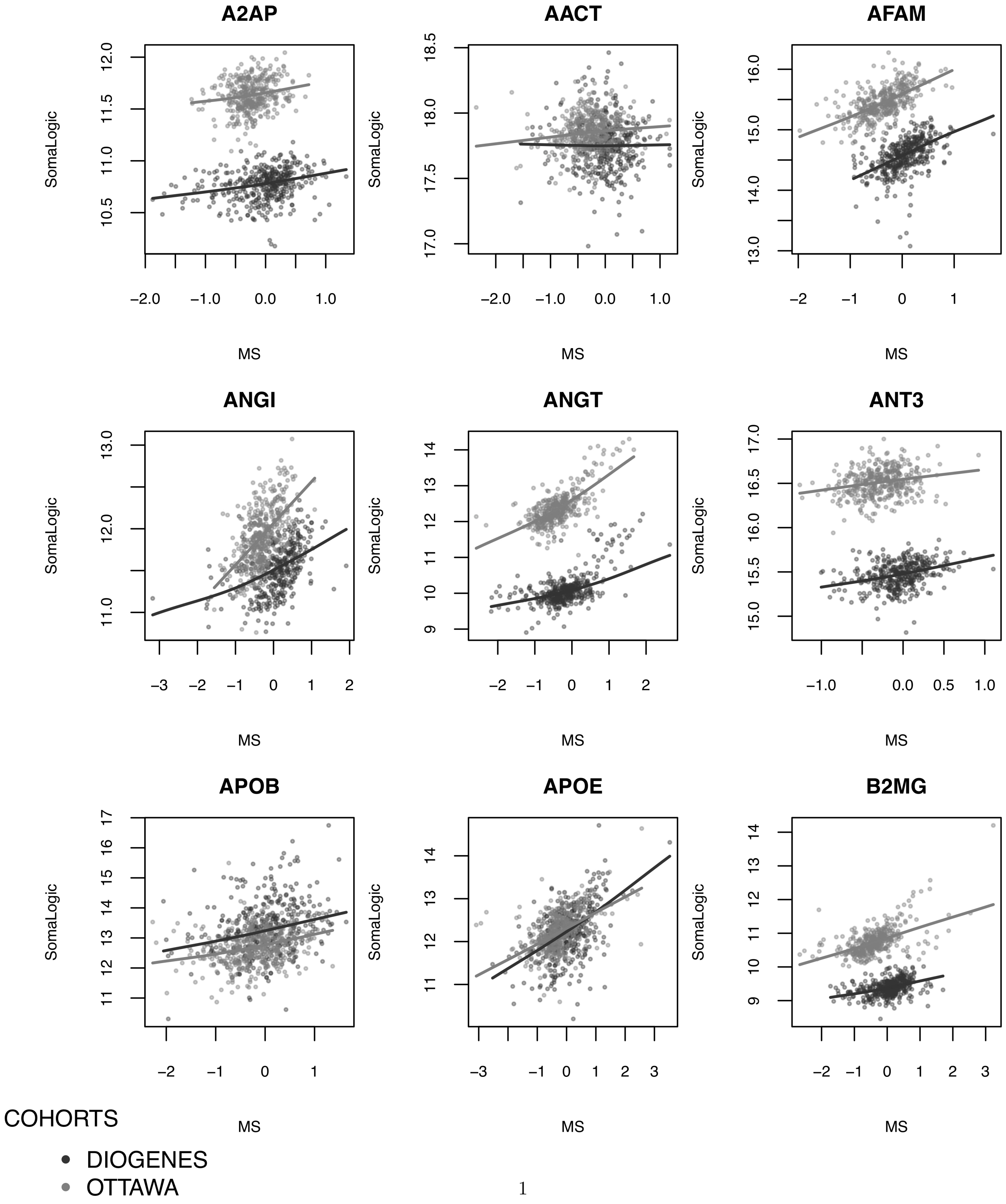

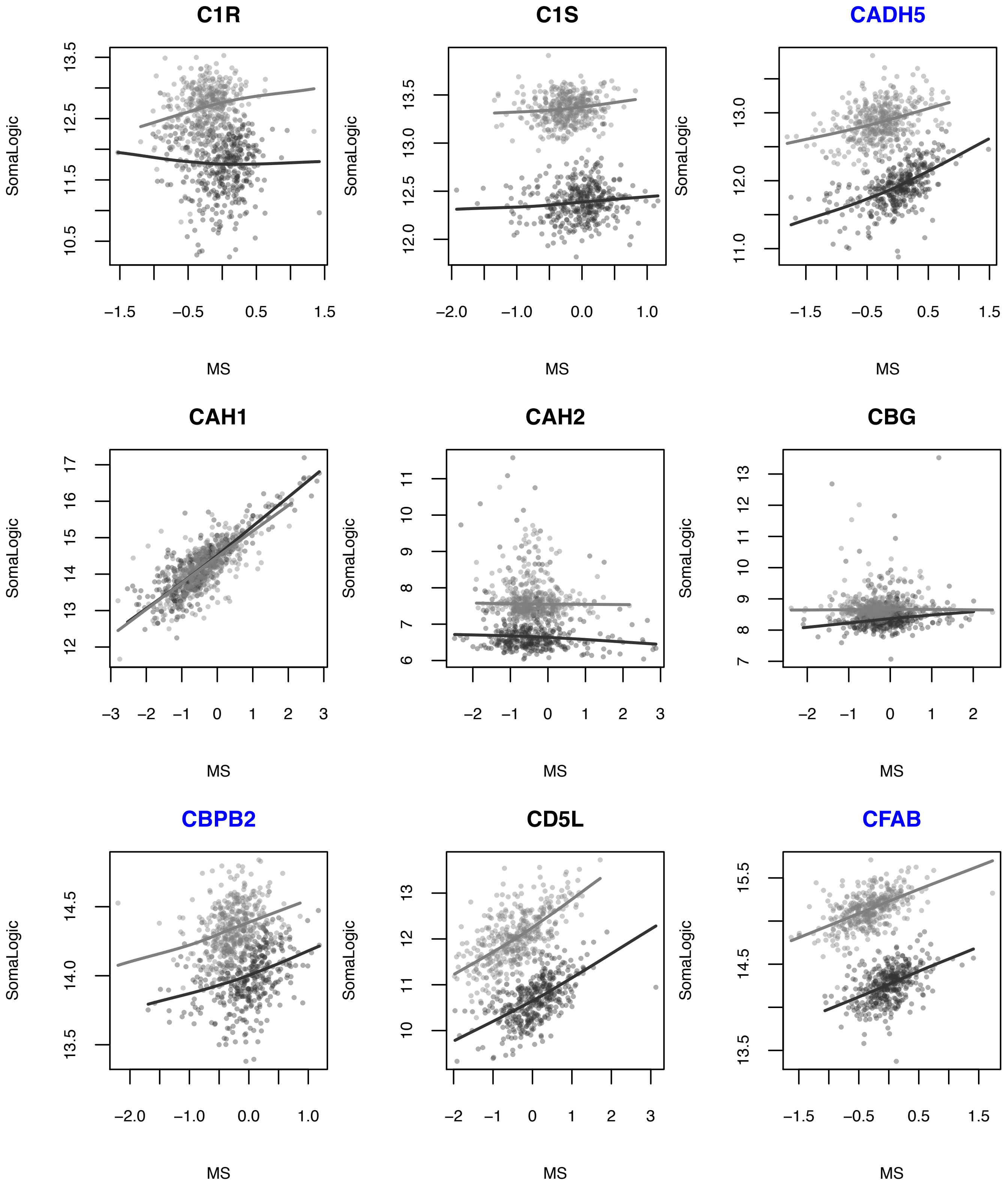
C1R
C1S
CADH5
CAH1
CAH2
CBG
CBPB2
CD5L
CFAB
SomaLogic
MS

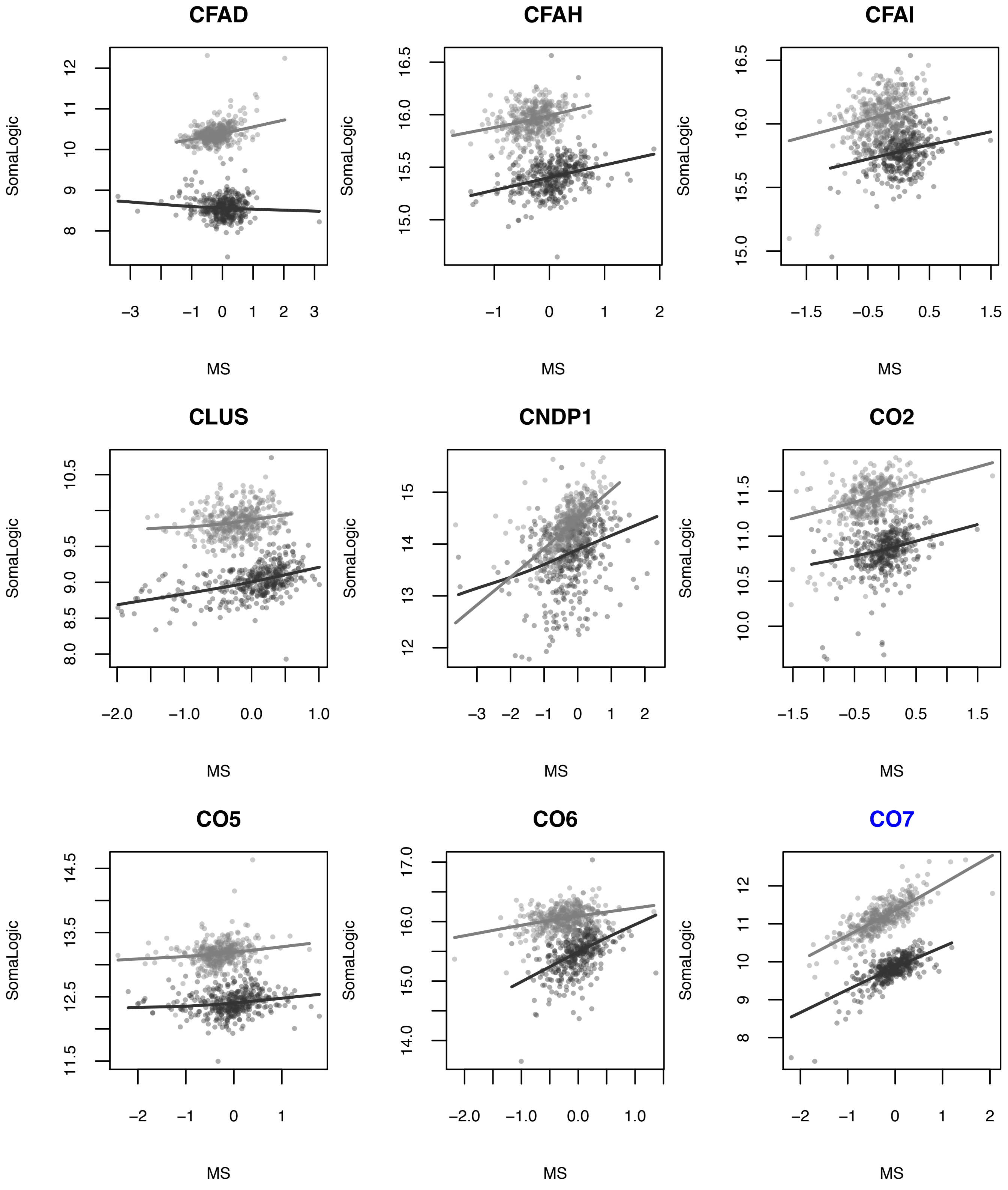
CFAD
CFAH
CFAI
CLUS
CNDP1
CO2
CO5
CO6
CO7
SomaLogic
MS

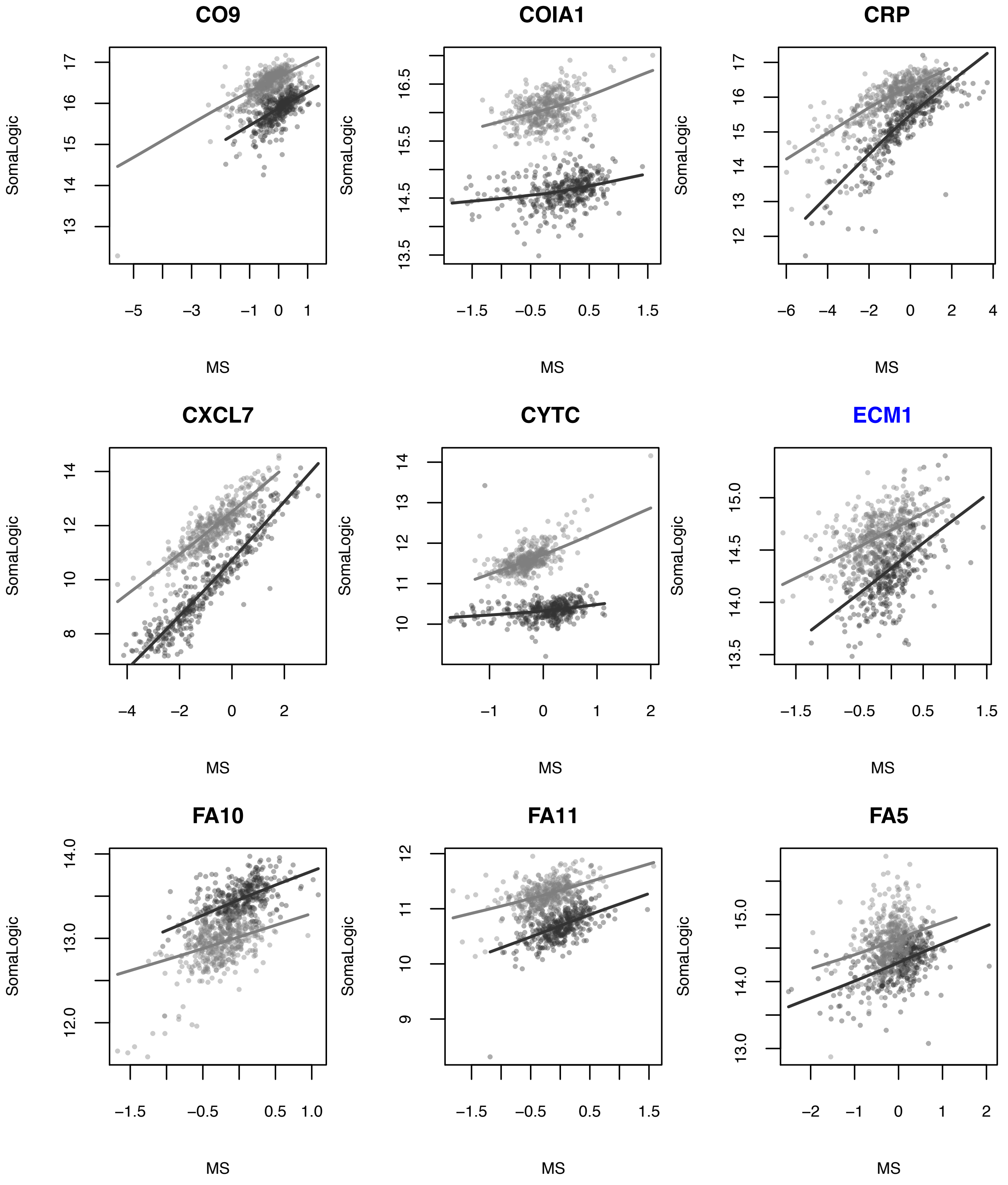
CO9
SomaLogic
13 14 15 16 17
−5 −3 −1 0 1
MS
COIA1
SomaLogic
13.5 14.5 15.5 16.5
−1.5 −0.5 0.5 1.5
MS
CRP
SomaLogic
12 13 14 15 16 17
−6 −4 −2 0 2 4
MS
CXCL7
SomaLogic
8 10 12 14
−4 −2 0 2
MS
CYTC
SomaLogic
10 11 12 13 14
−1 0 1 2
MS
ECM1
SomaLogic
13.5 14.0 14.5 15.0
−1.5 −0.5 0.5 1.5
MS
FA10
SomaLogic
12.0 13.0 14.0
−1.5 −0.5 0.5 1.0
MS
FA11
SomaLogic
9 10 11 12
−1.5 −0.5 0.5 1.5
MS
FA5
SomaLogic
13.0 14.0 15.0
−2 −1 0 1 2
MS

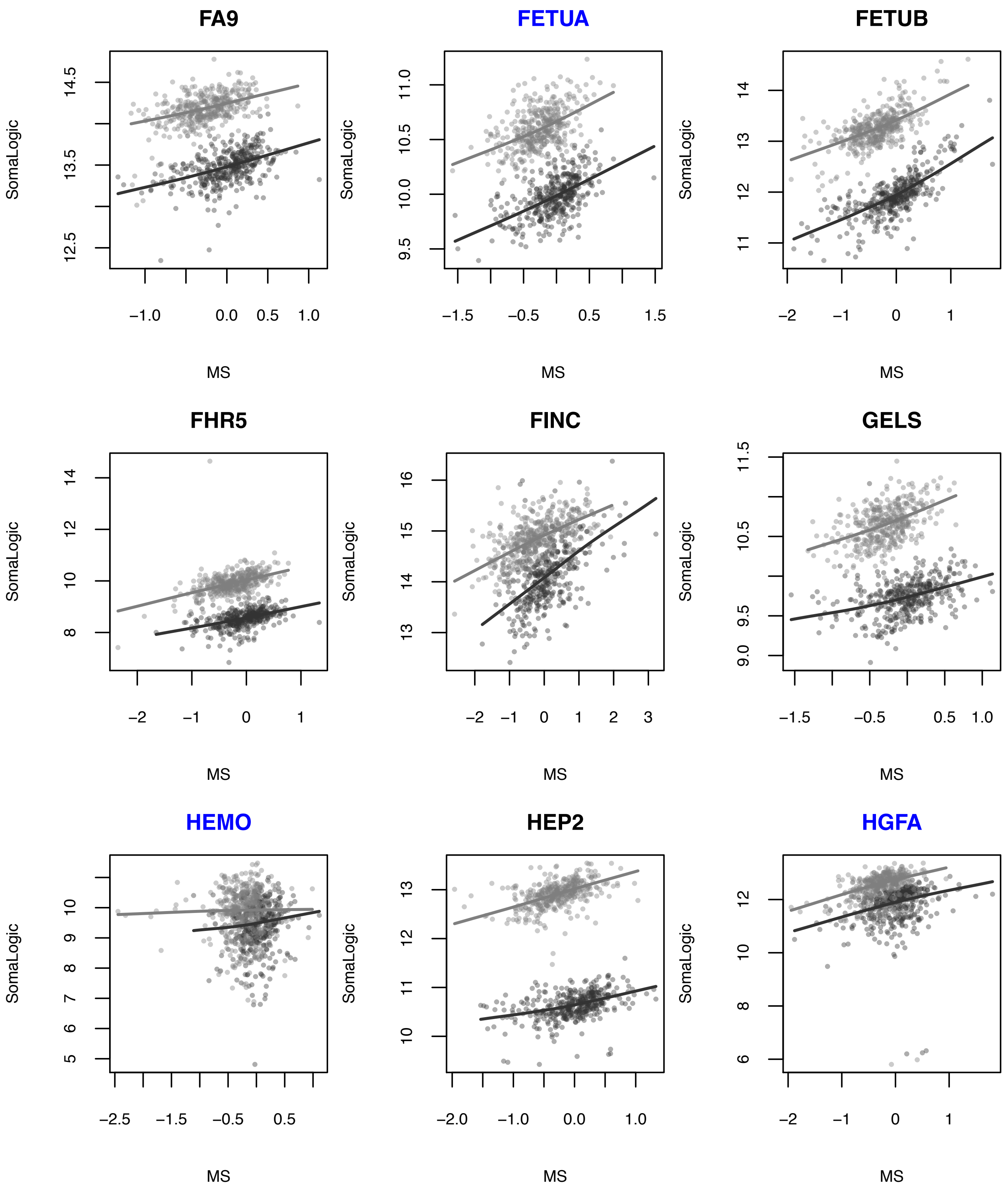
FA9
FETUA
FETUB
FHR5
FINC
GELS
HEMO
HEP2
HGFA
SomaLogic
MS

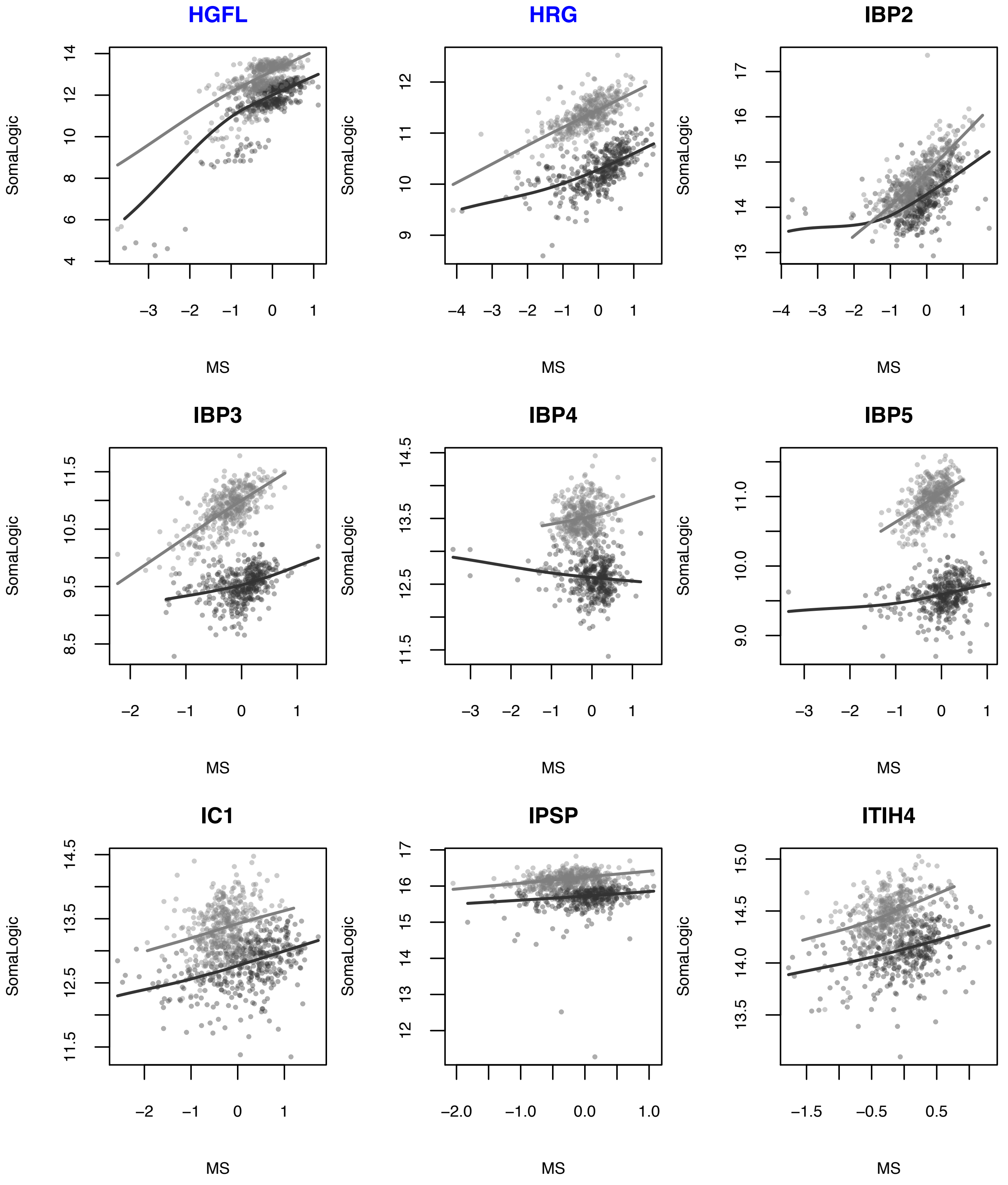
HGFL
SomaLogic
4
6
8
10
12
14
−3
−2
−1
0
1
MS
HRG
SomaLogic
9
10
11
12
−4
−3
−2
−1
0
1
MS
IBP2
SomaLogic
13
14
15
16
17
−4
−3
−2
−1
0
1
MS
IBP3
SomaLogic
8.5
9.5
10.5
11.5
−2
−1
0
1
MS
IBP4
SomaLogic
11.5
12.5
13.5
14.5
−3
−2
−1
0
1
MS
IBP5
SomaLogic
9.0
10.0
11.0
−3
−2
−1
0
1
MS
IC1
SomaLogic
11.5
12.5
13.5
14.5
−2
−1
0
1
MS
IPSP
SomaLogic
12
13
14
15
16
17
−2.0
−1.0
0.0
1.0
MS
ITIH4
SomaLogic
13.5
14.0
14.5
15.0
−1.5
−0.5
0.5
MS

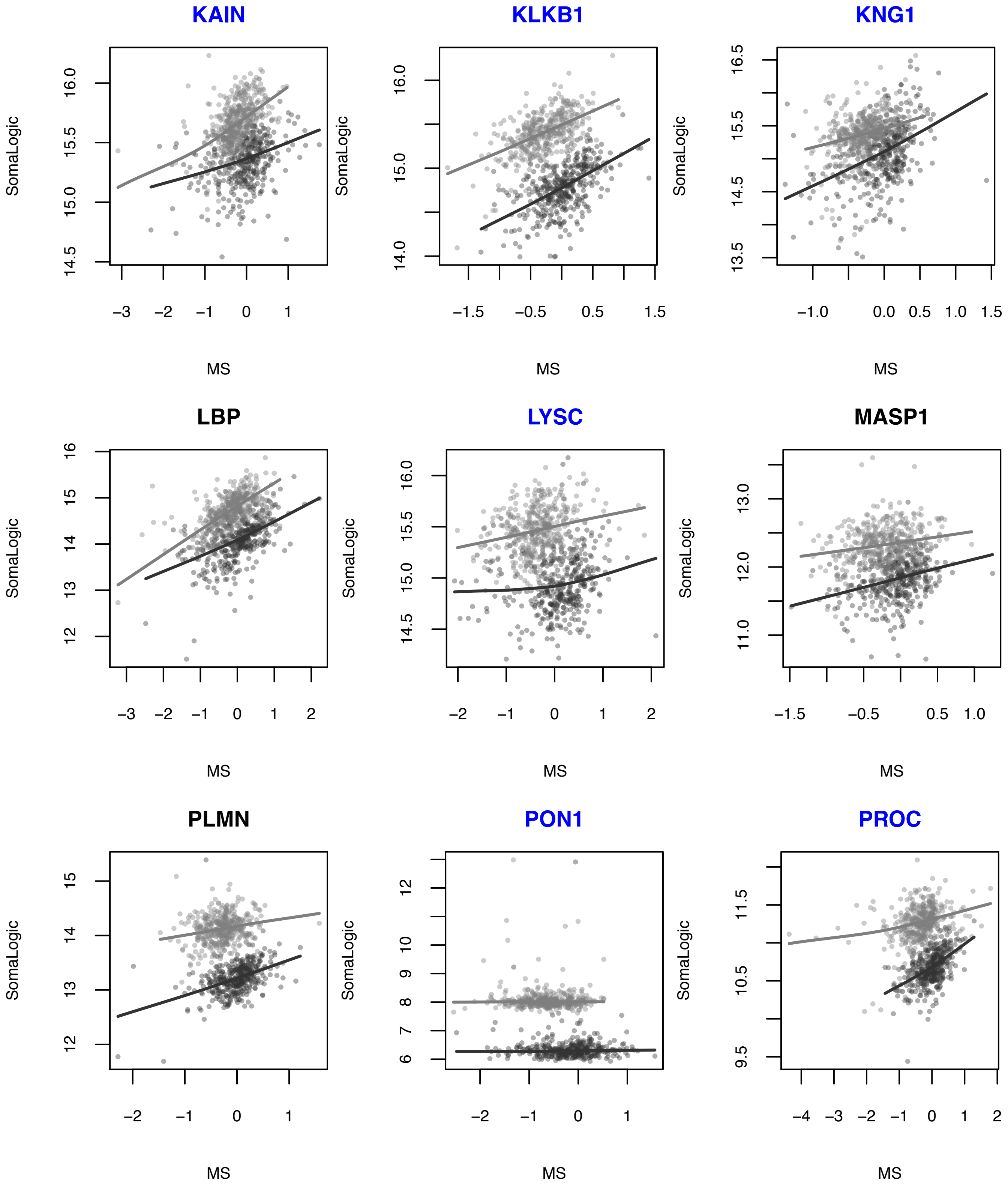
KAIN
KLKB1
KNG1
LBP
LYSC
MASP1
PLMN
PON1
PROC
SomaLogic
MS
14.5 15.0 15.5 16.0
−3 −2 −1 0 1
14.0 15.0 16.0
−1.5 −0.5 0.5 1.5
13.5 14.5 15.5 16.5
−1.0 0.0 0.5 1.0 1.5
12 13 14 15 16
−3 −2 −1 0 1 2
14.5 15.0 15.5 16.0
−2 −1 0 1 2
11.0 12.0 13.0
−1.5 −0.5 0.5 1.0
12 13 14 15
−2 −1 0 1
6 7 8 9 10 12
−2 −1 0 1
9.5 10.5 11.5
−4 −3 −2 −1 0 1 2

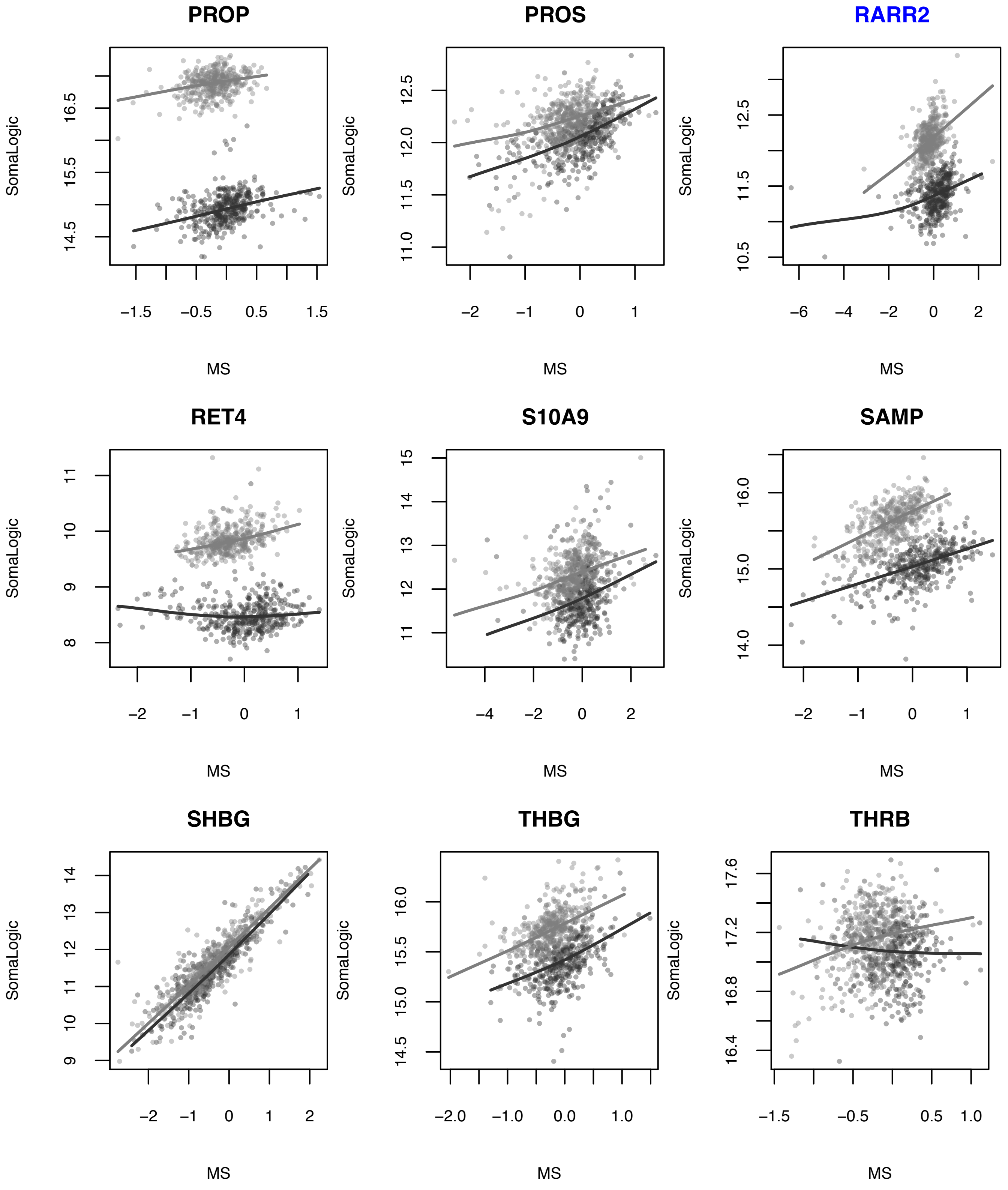
PROP
SomaLogic
MS
14.5
15.5
16.5
−1.5
−0.5
0.5
1.5
PROS
SomaLogic
MS
11.0
11.5
12.0
12.5
−2
−1
0
1
RARR2
SomaLogic
MS
10.5
11.5
12.5
−6
−4
−2
0
2
RET4
SomaLogic
MS
8
9
10
11
−2
−1
0
1
S10A9
SomaLogic
MS
11
12
13
14
15
−4
−2
0
2
SAMP
SomaLogic
MS
14.0
15.0
16.0
−2
−1
0
1
SHBG
SomaLogic
MS
9
10
11
12
13
14
−2
−1
0
1
2
THBG
SomaLogic
MS
14.5
15.0
15.5
16.0
−2.0
−1.0
0.0
1.0
THRB
SomaLogic
MS
16.4
16.8
17.2
17.6
−1.5
−0.5
0.5
1.0
