# Supplementary material for: A fully joint Bayesian quantitative trait locus mapping of human protein abundance in plasma
Source: PLoS Comput Biol. 2020 Jun 3;16(6):e1007882. doi: 10.1371/journal.pcbi.1007882 (PMC7295243; doi:10.1371/journal.pcbi.1007882)
Supplement: S5 Appendix — Detailed description of the quality control performed on the genotyping data for the Ottawa and DiOGenes studies. (PDF) [file pcbi.1007882.s005.pdf]

S5 Appendix to:
“A fully joint Bayesian quantitative trait locus mapping of human protein abundance in plasma”
Addendum to the genotyping quality control

The same quality control steps have been applied to the Ottawa and DiOGenes genotyping datasets. In the Ottawa study, $2,418$ subjects had genotyping data available and 80 were discarded for quality reasons (3.3%). In the DiOGenes study, 833 subjects had genotyping data available and 25 (3%). The numbers of subjects removed by the filters are detailed in Table A. These numbers concern subjects with genotyping data; only a subset of them have proteomic data available and could be used for our pQTL analyses, see Material and methods.

Filter	Ottawa study	DiOGenes study
Low call rate ($< 95\%$)	7	2
Abnormal autosomal heterozygosity	28	11
XXY karyotype	2	1
Gender discrepancies	41	9
High IBS	11	6

Table A: Number of subjects discarded by the different quality filters for the Ottawa and DiOGenes studies. A same subject can be flagged by several filters.
